# Targeting Vulnerable Plaques in Coronary Artery Disease: Detecting Risk, Preventing Events

**DOI:** 10.1007/s11886-025-02302-y

**Published:** 2025-11-27

**Authors:** Ryota Kakizaki, Sarah Bär, Lorenz Räber

**Affiliations:** Department of Cardiology, Bern University Hospital, Inselspital, University of Bern, Freiburgstrasse 18 , Bern, 3010 Switzerland

**Keywords:** Optical coherence tomography, Intravascular ultrasound, Near-infrared spectroscopy, Coronary computed tomography angiography, Lipid-lowering therapy, Inflammation

## Abstract

**Purpose of Review:**

To summarize the key features of vulnerable plaques identified by intracoronary and non-invasive imaging and explore how anti-atherosclerotic therapies contribute to plaque regression and stabilization.

**Recent Findings:**

Compared with high-dose statin therapy, intensive lipid-lowering with proprotein convertase subtilisin/kexin type 9 inhibitors achieves lower low-density lipoprotein cholesterol (LDL-C) levels and results in greater plaque regression and fibrous cap thickening. Recent data suggest that preventive intervention for functionally non-significant but high-risk plaques may reduce clinical events, and this strategy is now being further evaluated in randomized controlled trials.

**Summary:**

Detecting and treating vulnerable plaques is essential for improving the prognosis of patients with coronary artery disease. The extent of plaque volume reduction and fibrous cap thickening appears closely associated with on-treatment LDL-C levels. Anti-inflammatory therapies may provide additional stabilizing effects. Preventive treatment of high-risk, non-obstructive lesions and the use of non-invasive imaging to identify vulnerable plaques and high-risk individuals are promising strategies warranting further research.

## Introduction

Treatment of coronary artery disease (CAD) has long focused on targeting coronary stenosis through stenting or coronary artery bypass grafting. While these strategies reduce mortality and morbidity in acute coronary syndrome (ACS), no mortality benefit was shown among patients with chronic coronary syndrome [1]. This may reflect the ongoing emphasis on stenotic and ischemia-inducing lesions, overlooking that a large proportion of acute myocardial infarctions (AMI) arise from vulnerable plaques, i.e. the precursors to rupture or erosion.

Pathological studies show that ruptured plaques typically contain lipid-rich necrotic cores with thin fibrous caps, abundant macrophages and lymphocytes, reduced smooth muscle cells, and expansive remodeling—features that define thin-cap fibroatheroma (TCFA) [2–4]. A “thin” cap is defined pathologically as < 65 μm, based on morphometric analysis of 41 ruptured plaques, 95% of which had caps < 64 μm [4].

Although plaque erosion accounts for ~ 30% of ACS cases, its precursor features and in vivo imaging characteristics are still not well established. Eroded plaques are pathologically rich in proteoglycans and smooth muscle cells and often have thick fibrous caps despite lipid cores. However, current imaging techniques remain limited in detecting these features.

Taken together, vulnerable plaques are pathologically characterized by advanced atherosclerosis with high plaque burden (PB), lipid-rich necrotic cores, and thin fibrous caps—hallmarks of lesions at increased thrombotic risk.

## Detection of Vulnerable Plaques by Intracoronary Imaging

Catheter-based intracoronary imaging enables morphological and compositional assessment of the coronary arterial wall to foster our understanding of this complex disease. In clinical practice, detection of vulnerable plaques primarily relies on intravascular ultrasound (IVUS), near-infrared spectroscopy (NIRS), and optical coherence tomography (OCT). Virtual histology IVUS (VH-IVUS) analyzes radiofrequency data from raw backscattered ultrasound signals to generate tissue composition maps; however, it is no longer used clinically due to limited accuracy. Each modality has distinct strengths and limitations, and no single technique offers a comprehensive evaluation of plaque volume, lipid burden, and fibrous cap thickness (FCT). Table 1 summarizes previous studies examining vulnerable plaque features. Many studies have used intracoronary imaging to identify plaque characteristics linked to adverse clinical outcomes. Among these, key high-risk features include minimum lumen area (MLA), PB, lipid burden, and FCT.

**Table 1:** Summary of clinical trials investigating impact of vulnerable features

Study	Enrolled patient	Analyzed patient	Analyzed lesion	Inclusion	Follow-up duration	Vulnerable feature	Frequency of lesions	Primary endpoint	Primary results
VH-IVUS									
PROSPECT [74]	697	623	2709	ACS	3 years	VH-TCFA	596 (22%)	MACE	HR 3.90, 95%CI 2.25–6.76, *p* < 0·001
		673	3160			PB ≥ 70%	283 (9%)		HR 8.72, 95%CI 5.13–14.82, *p* < 0·001
		673	3160			MLA ≤ 4.0mm^2^	620 (20%)		HR 5.00, 95%CI 2.94–8.51, *p* < 0·001
VIVA [75]	170	167	931	SAP or ACS	3 year	VH-TCFA	561 (60%)	Non-restenotic MACE	HR 7.53, 95%CI 1.12–50.55, *p* = 0.038
						PB > 70%	176 (19%)		HR: 8.13, 95%CI:1.63–40.56, *p* = 0.011
Atheroremo- IVUS [76]	581	581	724	SAP or ACS	1 year	VH-TCFA	271 (37%)	non-culprit lesion related MACE	HR 1.96, 95%CI 1.08–3.53, *p* = 0.026
						PB ≥ 70%	127 (21%)		HR 3.15, 95%CI 1.75–5.68, *p* < 0·001
						MLA ≤ 4.0mm^2^	206 (29%)		HR 1.36, 95%CI 0.74–2.48, *p* = 0.32
NIRS-IVUS									
LRP[5]	1563	1271	5755	CCS	2 year	maxLCBI_4mm_ > 400	664 (11%)	MACE	HR 4.22, 95%CI 2.39–7.45, *p* < 0·0001
PROSPECT II [6]	902	898	3500	Recent MI	4 year	maxLCBI_4mm_ ≥ 324.7	851 (24%)	Non-culprit lesion-related MACE	aHR 7.83, 95%CI 4.12–14.89, *p* < 0·0001
			3629			PB ≥ 70%	787 (22%)		aHR 12.94, 95%CI 6.36–26.32, *p* < 0·0001
			3629			MLA ≤ 4.0mm^2^	1375 (38%)		aHR 4.97, 95%CI 2.59–9.53, *p* < 0·0001
OCT									
CLIMA [10]	1003	1003	1776	SAP or ACS	1 year	MLA < 3.5mm^2^	702 (40%)	Clinical endpoint (CD and target LAD segment MI)	HR 6.12, 95%CI 2.1–18.1, *P* < 0.001
						minFCT < 75 μm	323 (18%)		
						lipid angle > 180°	726 (41%)		
						macrophage	1021 (58%)		
COMBINE FFR-OCT [12]	550	390	445	SAP or ACS	1.5 year	minFCT ≤ 65 μm	104 (23%)	investigated lesion-related MACE	HR 5.12, 95%CI 2.12–12.34, *p* < 0.001
PECTUS-OBS [13]	438	420	494	AMI	2 year	lipid angle ≥ 90	154 (31.2%)	MACE	HR 1.93, 95%CI 1.08–3.47, *p* = 0.02
						minFCT < 65	136 (27.5%)		
						plaque rupture	45 (9.1%)		
						thrombus	34 (6.9%)		
Xing et al. [14]	1474	1474		SAP or ACS	2 year	lipid angle > 90	536	Non-culprit MACE	HR 1.975, 95%CI 1.046–3.731, *p* = 0.033
Jiang et al. [15]	883	883	3757	SAP or ACS	4 year	minFCT < 65 μm	515 (14%)	Composite endpoint	a HR 3.05, 95%CI 1.67–5.57, *p* < 0.001
						MLA < 3.5 mm^2^	1535 (41%)		aHR 3.71, 95%CI 1.22–11.34, *p* = 0.021
Kubo et al. [16]	1378	1378	3533	CCS and ACS	6 year	lipid angle > 180	567 (16%)	subsequent lesion-specific ACS events	HR 12.67, 95%CI 6.82–23.57, *p* < 0.001
						minFCT < 65 μm	163 (5%)		HR 10.41, 95%CI 6.48–16.73, *p* < 0.001
CCTA									
SCOT-HEART [58]	1778	1769	26,535	Suspected CCS	5 years	LAP	213 (0.8%)	CHD death or non-fatal MI	HR 1.60, 95%CI 0.67–3.80, *p* = 0.289
						Positive remodeling	1163 (4.4%)		HR 3.05, 95%CI 1.63–5.71, *p* < 0.001
						Napkin-ring sign	78 (0.3%)		HR 1.16, 95%CI 0.28–4.79, *p* = 0.841
						Spotty calcification	472 (1.8%)		HR 0.83, 95%CI 0.35–1.97, *p* = 0.669
						LAP or positive remodeling	1189 (4.5%)		HR 3.01, 95%CI 1.61–5.63, *p* = 0.001
PROMISE [59]	4996	4415	77,868	Suspected CCS	25 months	LAP	222 (5%)	MACE	NA
						Positive remodeling	628 (14%)		NA
						Napkin ring sign	169 (4%)		NA
						≥ 1 HRP feature	676 (15%)		HR 2.73, 95%CI 1.89–3.93
						≥ 2 HRP features	258 (6%)		HR 3.26, 95%CI 2.06–5.15
						3 HRP features	85 (2%)		HR 2.73, 95%CI 1.27–5.84
ICONIC [60]	25,251	468	NA	CCTA for evaluation of CAD	3.4 years	LAP	165 (35%)	ACS	aHR 1.38, 95%CI 1.051–1.805, *p* = 0.020
						Positive remodeling	392 (84%)		aHR 1.40, 95%CI 0.96–2.06, *p* = 0.085
						Spotty calcification	119 (25%)		aHR 1.54, 95%CI 1.17–2.04, *p* = 0.002
						≥ 2 HRP features	200 (43%)		aHR 1.59, 95%CI 1.22–2.08, *p* = 0.001

### NIRS-IVUS

The hybrid NIRS-IVUS catheter enables simultaneous imaging with NIRS and IVUS. IVUS uses ultrasound to visualize the vessel wall with a penetration depth of approximately 4–8 mm, allowing quantification of PB and assessment of arterial remodeling. However, its limited spatial resolution (~ 50 μm) makes it unsuitable for accurate FCT measurement. NIRS identifies lipid-rich plaques by analyzing unique light spectra generated by photon interactions with plaque components. The lipid core burden index (LCBI) is calculated by multiplying the proportion of valid pixels with a lipid probability > 0.6 by 1,000.

In the Lipid-Rich Plaque (LRP, NCT02033694) study, NIRS-IVUS was performed in two non-culprit coronary arteries of 1,271 patients undergoing percutaneous coronary intervention (PCI). Lesion-level analysis showed that a maximum LCBI in a 4 mm segment (maxLCBI_4mm_) > 400 independently predicted non-culprit major adverse cardiovascular events (MACE) at 24 months (adjusted hazard ratio [aHR]: 3.39; 95% confidence interval [CI]: 1.84–6.20; *p* < 0.0001) [5]. In A Multicentre Prospective Natural History Study Using Multimodality Imaging in Patients With ACS (PROSPECT II, NCT02171065) study, NIRS-IVUS was performed in three major coronary arteries of patients after MI. Lesion-level analysis showed that both maxLCBI_4mm_ ≥ 324.7 (aHR: 3.80; 95%CI: 1.87–7.70; *p* = 0.0002) and PB ≥ 70% (aHR: 5.37; 95%CI: 2.42–11.89; *p* < 0.0001) were independently associated with non-culprit lesion–related MACE over four years, while MLA ≤ 4.0 mm² only tended to be predictor (aHR: 1.85; 95%CI: 0.95–3.61; *p* = 0.072). Notably, lesions with neither maxLCBI_4mm_ ≥ 400 nor plaque burden ≥ 70% had an extremely low 4-year MACE rate of 0.2% [6].

### OCT

OCT employs near-infrared light and offers resolution approximately 10–20 times higher than IVUS (10–15 μm), enabling more accurate lumen measurements [7] and detailed plaque morphology evaluation including FCT assessment. However, limited tissue penetration—especially in fibroatheromas and thrombotic lesions—can hinder accurate assessment of vessel area. A FCT < 65 μm is the widely accepted threshold for defining TCFA, based on histological studies [8]. However, due to tissue shrinkage during histological processing, a threshold of 75–80 μm is also considered [9].

In the long-term substudy of the Relationship Between Coronary Plaque Morphology of Left Anterior Descending Artery and Long Term Clinical Outcome (CLIMA, NCT02883088) study [10], the presence of four high-risk OCT features—MLA < 3.5 mm², minimum FCT < 75 μm, lipid angle > 180°, and macrophage accumulation—was independently associated with 5-year cardiac death and target left anterior descending artery segment MI (HR: 5.16; 95%CI: 2.64–10.06; *p* < 0.001). TCFA was also independently associated with the same endpoint (HR: 3.46; 95%CI: 2.17–5.53; *p* < 0.001). Interestingly, no significant improvement in the predictive performance was observed between the individual presence of TCFA and the combination of four high-risk OCT features [11]. The Combined Optical Coherence Tomography Morphologic and Fractional Flow Reserve Hemodynamic Assessment of Non-Culprit Lesions to Better Predict Adverse Event Outcomes in Diabetes Mellitus Patients (COMBINE FFR-OCT, NCT02989740) study assessed 445 functionally non-significant lesions in 388 diabetic patients with stable or unstable CAD, showing that TCFA predicted investigated lesion-related MACE at 18 months (HR: 5.12; 95%CI: 2.12–12.34; *p* < 0.001) [12]. In the Identification of Risk Factors for Acute Coronary Events by OCT After STEMI and NSTEMI in Patients With Residual Non–flow Limiting Lesions (PECTUS-obs, NCT03857971) study of 494 lesions in non-infarct-related arteries (non-IRAs) from 438 AMI patients, OCT-derived high-risk features—maximum lipid angle ≥ 90°, minimum FCT < 65 μm, or the presence of plaque rupture/thrombus—were associated with MACE (HR: 1.93; 95%CI: 1.08–3.47; *p* = 0.02), and remained independent predictors in multivariable analysis [13]. In a study by Xing et al., OCT assessment of non-culprit lesions in culprit vessels of 1,474 AMI patients found lipid-rich plaque predicted non-culprit MACE over 24 months (HR: 1.975; 95%CI: 1.046–3.731; *p* = 0.033) [14]. In a three-vessel OCT study by Jiang et al. with 883 AMI patients, TCFA and MLA < 3.5 mm² both independently predicted lesion-level events. Lesions with both had markedly higher cumulative events over 3.3 years (aHR: 15.50; 95%CI: 6.89–34.89; *p* < 0.001) [15]. In another study by Kubo et al., OCT of 3,533 non-culprit plaques in 1,378 patients showed no events arose from fibrous/fibrocalcific plaques over 6 years, while 72 events originated from 1,909 lipid plaques. Maximum lipid angle > 180° (HR: 12.67; 95%CI: 6.82–23.57; *p* < 0.001) and minimum FCT < 65 μm (HR: 10.41; 95%CI: 6.48–16.73; *p* < 0.001) were both significantly associated with future ACS [16].

Collectively, MLA ≤ 4.0 mm² (IVUS) or ≤ 3.5 mm² (OCT), PB > 70%, maxLCBI_4mm_ ≥ 325, maximum lipid angle > 180°, minimum FCT ≤ 75 μm, macrophage accumulation, and thrombus are hallmark vulnerable plaque features. However, not all vulnerable plaques cause ACS or death. Many thrombi remain subclinical and organize into layered plaques, contributing to silent progression. This may explain why most events in imaging studies involve unstable angina or revascularization rather than MI or death. While the negative predictive value of lipid rich plaques is consistently > 95%, the positive predictive value remains limited to 10–20%.

## Effects of Lipid-Lowering Therapy on Coronary Plaque Regression and Stabilization

Lipid-lowering therapy improves outcomes in atherosclerotic CAD, with lower achieved low-density lipoprotein cholesterol (LDL-C) levels linked to better prognosis [17, 18]. Serial coronary imaging suggests plaque regression and stabilization as primary mechanisms. Table 2 summarizes studies examining these effects.

**Table 2:** Summary of clinical trials investigating Lipid-Lowering therapies and plaque regression/stabilization

Study	Patient	Inclusion	Lesion severity	FU duration	Treatment arm	LDL-C Change	Modality	Imaging endpoint	Analyzed vessel	Change
Statin										
REVERSAL [19]	657	CCS	20–50%	18 month	Atorvastatin 80 mg/day	−71.1	IVUS	TAV	253	−0.9 (−3.5 to 1.6)
								PAV		0.2 (−0.3 to 0.5)
					Pravastatin 40 mg/day	−39.8		TAV		2.7 (0.2 to 4.7)
								PAV		1.6 (1.2 to 2.2)
ASTEROID [20]	507	SAP or UAP	20–50%	24 month	Rosuvastatin 40 mg/day	−53.2	IVUS	PAV	349	−0.79 (−1.21 to −0.53)
								normalized TAV		−12.5 (−15.08 to −10.48)
Saturn [21]	1578	CCS	20–50%	104 week	Rosuvastatin 40 mg/day	−57.4	IVUS	PAV	520	−1.22 (1.52 to −0.90)
								normalized TAV		−6.39 (−7.52 to −5.12)
					Atorvastatin 80 mg/day	−49.7		PAV	519	−0.99 (−1.19 to −0.63)
								normalized TAV		−4.42 (−5.98 to −3.26)
EASY-FIT [77]	70	UAP	30–70%	12 month	Atorvastatin 20 mg/day	−58	OCT	minFCT	30	73 (23 to 113)
								max lipid angle		−50 (−60 to −30)
					Atorvastatin 5 mg/day	−44		minFCT	30	19 (−1 to 48)
								max lipid angle		−10 (−20 to −0.1)
IBIS-4 [22, 23]	103	STEMI	< 50%	13 month	High-dose statin	−54.4	OCT	minFCT	31	24.41 (6.84 to 41.98)
								mean macrophage angle	153	−3.22 (−4.94 to −1.51)
						−54.1	IVUS	PAV	146	−0.9 (−1.56 to −0.25)
								normalized TAV		−12.18 (−16.91 to −7.44)
YELLOW [24]	87	SAP	> 70%	6–8 week	Rosuvastatin 40 mg/day	−20.7	NIRS	maxLCBI_4mm_	36	−149.1 (−210.9 to −42.9)
					Standard therapy	−0.9		maxLCBI_4mm_	34	2.4 (−36.1 to 44.7)
YELLOW II [25]	91	CCS	NA	8–12 week	Rosuvastatin 40 mg/day	−36.2	OCT	minFCT	85	9.1 ± 17.1
Auscher et al. [68]	96	AMI	NA	12 months	Rosuvastatin 40 mg/day	−73.5	CCTA	Total plaque volume	NA	43.5 ± 225.8
								PAV		−0.4 ± 3.2%
								Necrotic core volume		26.8 ± 122.1
					Simvastatin 40 mg/day or Atorvastatin 80 mg/day	−54.1		Total plaque volume		19.1 ± 190.2
								PAV		−0.6 ± 2.7
								Necrotic core volume		25.2 ± 80.9
Lo et al. [67]	37	HIV	< 70% < 50% in LM	12 months	Atorvastatin 20–40 mg/day	−38.7	CCTA	Non-calcified plaque volume	NA	−8.2 (−18.3 to 3.5)
					Placebo	+ 11.6				6.7 (−6.5 to 29.8)
REPRIEVE [66]	611	HIV	not clarified	24 months	Pitavastatin 4 mg/day	−30	CCTA	Non-calcified plaque volume	NA	−1.7 ± 25.2
					Placebo	+ 1				2.6 ± 27.1
Meta-analysis Andelius et al. [69]	792	CCS	not clarified	14.5 ± 9.5 months	Intensive statin	NA	CCTA	Total plaque volume	NA	−20.9 (−31.2 to −10.6)
					Moderate statin	NA		Total plaque volume		−1.7 (−10.0 to 6.7)
					Intensive or moderate statin	−65.1		LAP volume		−5.8 (−8.0 to −3.7)
								Non-calcified plaque volume		−7.6 (−17.4 to 2.1)
								Calcified plaque volume		11.8 (3.4 to 20.3)
					Controls	NA		Total plaque volume		15.0 (5.3 to 24.6)
								LAP volume		1.4 (−0.8 to 3.5)
								Non-calcified plaque volume		12.0 (−1.8 to 25.9)
								Calcified plaque volume		5.0 (−2.0 to 12.0)
Ezetimibe										
PRECISE-IVUS [26]	246	ACS or SAP	not clarified	9–12 month	Ezetimibe + statin	−40	IVUS	PAV	100	−1.4 (−3.4 to −0.1)
								TAV		−5.3 (−12.4 to 0.1)
					statin	−29		PAV	102	−0.3 (−1.9 to 0.9)
								TAV		−1.2 (−5.7 to 3.3)
PCSK9 inhibitor										
GLAGOV [29]	968	CCS	20–50%	72 weeks	Evolocumab + statin	−56.3	IVUS	PAV	423	−0.95 (−1.33 to −0.58)
								TAV		−5.80 (−8.19 to −3.41)
					Placebo + statin	0.2		PAV	423	0.05 (−0.32 to 0.42)
								TAV		−0.91 (−3.29 to 1.47)
PACMAN-AMI [30]	300	AMI	20–50%	52 weaks	Alirocumab + high-dose statin	−131.2	IVUS	PAV	263	−2.13 (−2.53 to −1.73)
								normalized TAV		−26.12 (−30.07 to −22.17)
							OCT	minFCT	173	62.67 (48.84 to 76.50)
								mean macrophage angle	232	−25.98 (−29.35 to −22.61)
							NIRS	maxLCBI_4mm_	252	−79.42 (−100.39 to −58.46)
					Placebo + high-dose statin	−76.5	IVUS	PAV	274	−0.92 (−1.28 to −0.56)
								normalized TAV		−14.97 (−18.14 to −11.80)
							OCT	minFCT	197	33.19 (22.22 to 44.16)
								mean macrophage angle	251	−15.95 (−19.02 to −12.87)
							NIRS	maxLCBI_4mm_	260	−37.60 (−57.40 to 17.80)
	164	NSTEMI	20–50%	50 weeks	Evolocumab + statin	−114.2	OCT	minFCT	80	42.7 (32.4 to 53.1)
								max lipid angle		−57.5 (−72.2 to −42.7)
					Placebo + statin	−55.3		minFCT	81	21.5 (10.9 to 32.1)
								max lipid angle		−31.4 (−50.2 to −12.7)
ODYSSEY J-IVUS [32]	206	ACS	> 50%	36 week	Alirocumab + statin	−63.2	IVUS	normalized TAV	93	−4.8 ± 1.0%
								PAV		−1.4 ± 0.4%
					Standard care	−15.5		normalized TAV	89	−3.1 ± 1.0%
								PAV		−1.3 ± 0.4%
Hirai et al. [71]	98	High-risk for ASCVD	not clarified	6 months	Evolocumab + statin	−51.1	CCTA	HU increase	136 plaques	From 39.1 ± 8.1 to 84.9 ± 31.4
								Change in remodeling index		From 1.29 ± 0.11 to 1.19 ± 0.10
					Statin	−11.9		HU increase		From 44.0 ± 6.0 to 44.0 ± 9.5
								Change in remodeling index		From 1.35 ± 0.12 to 1.34 ± 0.20
Low-dose colchicine										
COCOMO-ACS [78]	64	NSTEMI	20–50%	18 month	Colchicine 0.5 mg/day	−54.1	OCT	minFCT	28	37.2 ± 21.3
								max lipid angle		−54.8 ± 46.9
					Placebo	−50.3		minFCT	29	29.2 ± 20.9
								max lipid angle		38.8 ± 32.2
COLOCT [48]	128	ACS	30–70%	12 month	Colchicine 0.5 mg/day	−34.7	OCT	minFCT	110	87.2 (69.9 to 104.5)
								mean macrophage angle		–14.0 (–18.0 to − 10.0)
								MLA		–0.2 (–0.4 to − 0.1)
					Placebo	−19.3		minFCT	95	51.9 (32.8 to 71.0)
								mean macrophage angle		–8.9 (–13.3 to − 4.6)
								MLA		–0.3 (–0.5 to − 0.1)
Vaidya et al. [72]	80	Recent ACS < 1 month	not clarified	12.6 months	Colchicine 0.5 mg/day	−19.0	CCTA	LAP volume	NA	−15.9 ± 17.3
								Total plaque volume		−42.3 ± 66.6
					Standard care	−18.9		LAP volume		−6.6 ± 12.8
								Total plaque volume		−26.4 ± 42.1
Icosapent ethyl										
EVAPORATE [70]	68	CCS	≥ 20%	18 months	Icosapent ethyl 4 g/day + statin	−2.4	CCTA	LAP volume	NA	−0.3 ± 1.5*
								Total plaque volume		−0.5 ± 0.8*
								Non-calcified plaque volume		−0.8 ± 1.2*
								Calcified plaque volume		0.0 ± 0.5*
					Placebo + statin	−12.8		LAP volume		0.9 ± 1.7*
								Total plaque volume		0.4 ± 1.2*
								Non-calcified plaque volume		0.3 ± 1.3*
								Calcified plaque volume		0.4 ± 1.2*

### Statins

High-intensity and high-dose statins induce more plaque regression than lower-intensity or lower-dose statins. In the Reversal of Atherosclerosis with Aggressive Lipid Lowering (REVERSAL) trial, 502 patients underwent serial IVUS of coronary arteries with 20–50% stenosis over 18 months. Atorvastatin 80 mg/day led to smaller percent increases in total atheroma volume (TAV) than pravastatin 40 mg/day (–0.4% [–2.4 to 1.5] vs. 2.7% [0.2 to 4.7]; *p* = 0.02) [19]. In A Study to Evaluate the Effect of Rosuvastatin on Intravascular Ultrasound-Derived Coronary Atheroma Burden (ASTEROID, NCT00240318), 349 patients with stable or unstable angina received rosuvastatin 40 mg/day for 24 months. Percent atheroma volume (PAV) decreased significantly from 39.9 (33.8–45.3)% to 38.5 (32.6–44.3)% (–0.79 [−1.21 to −0.53]), with plaque regression seen in 63.6% of patients [20]. The Study of Coronary Atheroma by Intravascular Ultrasound: Effect of Rosuvastatin versus Atorvastatin (SATURN, NCT000620542) included 1,039 patients with 20–50% stenosis and followed them for 104 weeks. LDL-C was lower with rosuvastatin 40 mg/day than with atorvastatin 80 mg/day (62.6 ± 1.0 vs. 70.2 ± 1.0 mg/dL; *p* < 0.001), and normalized TAV decreased more with rosuvastatin (–6.39 mm³ [–7.52 to − 5.12] vs. − 4.42 mm³ [–5.98 to − 3.26]; *p* = 0.01) [21].

High-dose statins also stabilize plaque composition. The Integrated biomarker imaging study (IBIS-4, NCT00962416) simultaneously evaluated plaque regression and stabilization with high-dose statin therapy over 13 months. A total of 103 patients with ST-elevation myocardial infarction (STEMI) received rosuvastatin 40 mg/day, and serial VH-IVUS and OCT were performed in two non-IRAs with < 50% stenosis. In 82 patients with IVUS data, PAV decreased from 43.95 ± 9.66 to 43.02 ± 9.82% (–0.90% [−1.56 to −0.25]; *p* = 0.007) [22]. In 83 patients with OCT data, minimum FCT increased from 64.88 ± 19.89 to 87.88 ± 38.08 μm (+ 24.41 μm [6.84–41.98]; *p* = 0.008), and mean macrophage angle decreased from 9.63 ± 12.84° to 6.40 ± 9.64° (–3.22° [−4.94 to −1.51]; *p* < 0.001) [23].

Short-term effects of statins have also been studied. In the Reduction in Yellow Plaque by Intensive Lipid Lowering Therapy (YELLOW, NCT01567826) trial, 87 patients with functionally significant lesions underwent serial NIRS-IVUS. After 6–8 weeks, normalized TAV remained unchanged, but maxLCBI_4mm_ decreased significantly in the high-dose statin group (490.6 [363.8–689.7.8.7] to 336.1 [252.3–479.9.3.9]), while no meaningful change occurred in the standard group (356.7 [145.2–509.2.2.2] to 385.7 [139.2 to 510.9]) [24]. In the Reduction in Coronary Yellow Plaque, Lipids and Vascular Inflammation by Aggressive Lipid Lowering (YELLOW II, NCT01837823) study, involving 85 patients over 8–12 weeks, OCT showed increased minimum FCT (100.9 ± 41.7 to 108.6 ± 39.6 μm; *p* < 0.001), while NIRS-IVUS showed no change in maxLCBI₄mm (416.6 ± 172.9 to 400.2 ± 180.4; *p* = 0.52) or PAV (60.71 ± 7.52 to 60.97 ± 7.57%; *p* = 0.30) [25]. These results suggest plaque volume reduction may take longer than improvements in lipid burden or FCT.

### Ezetimibe

Although limited, existing evidence suggests that LDL-C reduction with ezetimibe contributes to plaque regression. In the Plaque Regression With Cholesterol Absorption Inhibitor or Synthesis Inhibitor Evaluated by Intravascular Ultrasound (PRECISE-IVUS, NCT01043380) trial, 202 patients were randomized to receive atorvastatin plus ezetimibe or atorvastatin alone. After 9–12 months, the combination group had a significantly greater reduction in PAV than the atorvastatin-alone group (–1.4% [–3.4 to − 0.1] vs. − 0.3% [–1.9 to 0.9], *p* = 0.001) [26].

### Proprotein Convertase Subtilisin/kexin Type 9 (PCSK9) Inhibitors

The introduction of PCSK9 inhibitors allowed for achieving substantially lower LDL-C levels as compared with statins and ezetimibe. These agents provide greater cardiovascular risk reduction [27, 28] and also promote plaque regression and stabilization. In the Global Assessment of Plaque Regression With a PCSK9 Antibody as Measured by Intravascular Ultrasound (GLAGOV, NCT01813422) trial, 846 patients on statins were randomized to evolocumab or placebo. After 76 weeks, PAV decreased significantly in the evolocumab group (− 0.95% [–1.33 to − 0.58]) but not in the placebo group (0.05% [–0.32 to 0.42]), with a between-group difference of − 1.0% (−1.8 to −0.64, *p* < 0.001) [29]. In the Effects of the PCSK9 Antibody Alirocumab on Coronary Atherosclerosis in Patients With Acute Myocardial Infarction (PACMAN-AMI, NCT03067844) trial, 300 AMI patients received alirocumab or placebo, and serial NIRS-IVUS and OCT imaging of non-IRAs with 20–50% stenosis over 52 weeks showed greater reductions in PAV (–1.21% [–1.78 to − 0.65]; *p* < 0.001) and maxLCBI₄mm (–41.2 [–70.7 to − 11.8]; *p* = 0.006), and greater increases in minimum FCT (+ 29.7 μm [11.8 to 47.6]; *p* = 0.001) with alirocumab compared to placebo [30]. Similarly, the High-Resolution Assessment of Coronary Plaques in a Global Evolocumab Randomized Study (HUYGENS, NCT03570697) trial randomized 135 NSTEMI patients to evolocumab or placebo. At 50 weeks, evolocumab significantly increased minimum FCT (21.2 [4.7 to 37.7], *p* = 0.015) and reduced maximum lipid angle (–26.0° [–49.6 to − 2.4]; *p* = 0.04) [31].

The short-term effects of PCSK9 inhibitors have also been studied. In the Effect of Evolocumab on Coronary Plaque Characteristics: a Multimodality Imaging Study (YELLOW III, NCT04710368), 110 stable angina patients with lipid-rich plaques received evolocumab for 26 weeks. OCT and NIRS showed significant increases in minimum FCT (70.9 ± 21.7 to 97.7 ± 31.1 μm; *p* < 0.001) and decreases in maxLCBI_4mm_ (306.8 ± 177.6 to 213.1 ± 168.0; *p* < 0.001). However, no significant short-term advantage of PCSK9 inhibitor over high-dose statins was observed. In the Evaluation of Effect of Alirocumab on Coronary Atheroma Volume in Japanese Patients Hospitalized for Acute Coronary Syndrome With Hypercholesterolemia (ODYSSEY J-IVUS, NCT02984982) study, 189 post-ACS patients were randomized to alirocumab or placebo. At 36 weeks, the percent change in normalized TAV did not differ significantly (difference: − 1.6%±1.4%; *p* = 0.228) [32]. The Functional Improvement of Non-infarcT relaTed Coronary Artery Stenosis by Extensive LDL-C Reduction With a PCSK9 Antibody (FITTER, NCT04141579) trial evaluated 142 patients with ACS and multivessel disease over 12 weeks of treatment with evolocumab or placebo. While maxLCBI_4mm_ decreased overall, no significant difference was observed between the groups. Changes in PAV and normalized TAV were also not statistically significant. These trials collectively suggest that PB and lipid burden reduction are processes that take at least 1 year time.

**Fig. 1:**
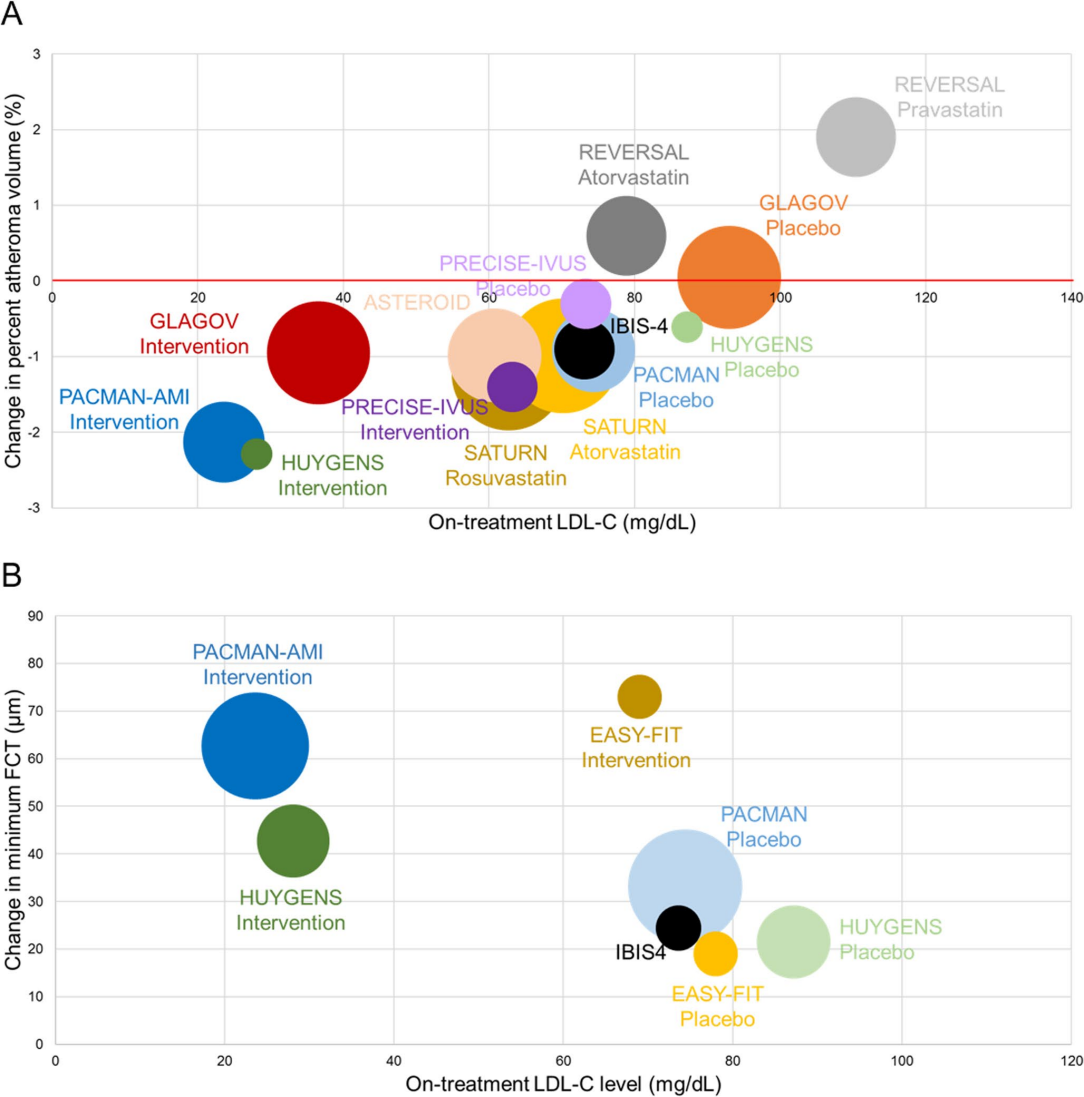
On-treatment Low-Density Lipoprotein Cholesterol Level and Changes in Coronary Plaque Characteristics across Trials. Bubble plot showing the change in percent atheroma volume (**Panel A**) and the change in minimum fibrous cap thickness (**Panel B**) (y-axis) in comparison to mean on-treatment low-density lipoprotein cholesterol level (x-axis). Bubble size is proportional to the overall number of patients

Figure 1 shows a bubble plot of on-treatment LDL-C levels versus absolute changes in PAV and minimum FCT. These data suggest that lower LDL-C is associated with greater plaque regression and fibrous cap thickening. However, rapid LDL-C lowering does not immediately lead to changes in plaque volume, lipid content, or FCT. These effects require time, even with intensive lipid-lowering therapy. Therefore, early initiation and long-term continuation of potent lipid-lowering treatment are essential for patients with acute coronary events and vulnerable plaques.

## Does Anti-inflammatory Therapy Promote Plaque Regression or Stabilization?

Inflammation plays a key role in all stages of atherosclerosis and remains a major contributor to residual cardiovascular risk. A pooled analysis of the three trials [33–35] showed that patients in the highest high-sensitivity C-reactive protein (hs-CRP) quartile had significantly higher MACE risk than those in the lowest quartile (aHR: 1.31; 95%CI: 1.20–1.43; *p* < 0.0001), despite statin therapy [36]. These findings support the role of anti-inflammatory therapy in ASCVD secondary prevention and its potential to promote plaque regression and stabilization.

### Low-dose Colchicine

Colchicine is an inexpensive, potent, and widely used anti-inflammatory drug. Its mechanisms include inhibition of microtubule formation, leukocyte motility, and cytokine release. It has long been used to treat gout, familial mediterranean fever, and pericarditis [37, 38]. Several RCTs have examined its cardiovascular benefits. A meta-analysis of six major trials [39–44]—including 21,800 patients, showed that low-dose colchicine significantly reduced MACE (HR: 0.75; 95%CI: 0.56–0.93), MI (HR: 0.71; 95%CI: 0.51–0.91), and urgent revascularization (HR: 0.67; 95%CI: 0.41–0.93) over 12 to 33.6 months [45]. Based on these data, the 2024 ESC Guidelines for chronic coronary syndromes and the 2025 ACC/AHA Guidelines for acute coronary syndromes upgraded low-dose colchicine to a Class IIa recommendation [46, 47]. However, the most recent CLEAR trial, including 7,062 post-MI patients, showed no significant difference between low-dose colchicine and placebo for MACE at a median 2.98-year follow-up (9.1% vs. 9.3%; HR: 0.99; 95%CI: 0.85–1.16; *p* = 0.93) [44]. Reasons for the discrepancy remain unclear. Higher rates of drug discontinuation (25.9%) and the impact of the COVID-19 pandemic may have contributed; however, on-treatment analyses and subgroup analyses across different phases of the pandemic yielded consistent results.

Intracoronary imaging studies have also assessed whether colchicine modifies plaque features. The Colchicine–Optical Coherence Tomography Trial (COLOCT, NCT04848857) randomized 128 AMI patients to colchicine or placebo, with OCT at baseline and 12 months for non-culprit lipid-rich plaques (30–70% stenosis). LDL-C reductions were comparable (*p* = 0.107), however, colchicine led to significantly greater minimum FCT increases (difference: +34.2 μm [9.7 to 58.6]; *p* = 0.006) [48].

While intensive lipid-lowering remains the cornerstone of fibrous cap thickening, it is unclear whether low-dose colchicine adds benefit regarding plaque vulnerability. Further studies are warranted to explore potential synergy between anti-inflammatory and lipid-lowering therapies for plaque stabilization.

### Biologic Anti-Cytokine Therapy and its Potential Effects on Coronary Plaques

Biologic agents targeting specific cytokines represent a promising strategy to enhance anti-inflammatory therapy beyond conventional approaches. Emerging data suggest these therapies may improve cardiovascular outcomes in high-risk patients. The Canakinumab Antiinflammatory Thrombosis Outcome Study (CANTOS, NCT01327846) enrolled 10,061 patients with prior MI and persistent systemic inflammation (hs-CRP ≥ 2 mg/L) despite optimal medical therapy (OMT). Patients were randomized to placebo or canakinumab—a monoclonal antibody targeting interleukin-1β (IL-1β)—every three months. The canakinumab 150 mg group had a lower rate of primary endpoint (nonfatal MI, nonfatal stroke, or cardiovascular death) compared to the placebo group (HR: 0.85; 95%CI: 0.74–0.98; *p* = 0.02075), meeting the significance threshold. All doses increased fatal infection/sepsis risk (0.31 vs. 0.18 events/100 person-years; *p* = 0.02) [49]. A CANTOS subanalysis showed that patients treated with canakinumab who achieved IL-6 levels below the median (1.65 ng/L) had a 32% lower MACE risk compared to placebo (aHR: 0.68; 95%CI: 0.56–0.82; *p* < 0.0001) [50], suggesting that IL-6 pathway modulation contributes to cardiovascular benefit. In A Phase 2b, Randomized, Double-Blind, Placebo-Controlled Trial to Evaluate Reduction in Inflammation in Patients With Advanced Chronic Renal Disease Utilizing Antibody Mediated IL-6 Inhibition (RESCUE, NCT03926117), ziltivekimab—a monoclonal antibody targeting the IL-6 ligand—achieved a 77–92% reduction in hs-CRP without inducing sustained grade 3 or 4 neutropenia or thrombocytopenia, which have been observed with other IL-6 inhibitors [51]. These findings support continued evaluation of ziltivekimab in cardiovascular populations. Ongoing RCTs include the ARTEMIS trial (NCT06118281) in patients with recent AMI, and the ZEUS trial (NCT05021835) in patients with chronic kidney disease and established ASCVD.

To date, there is no evidence evaluating whether anti-cytokine therapies affect atherosclerotic plaque volume or vulnerability. Our group aims to address this knowledge gap in the upcoming the Effects of Ziltivekimab on Coronary Atherosclerosis in Patients With Acute Myocardial Infarction (ZEPHYR) study, which will investigate the effects of ziltivekimab versus placebo on coronary plaques in non-IRAs of AMI patients using serial multimodality intracoronary imaging.

## Preventive PCI for Vulnerable Plaques

The role of preventive PCI for vulnerable, non-obstructive lesions is under active investigation. The Preventive Coronary Intervention on Stenosis with Functionally Insignificant Vulnerable Plaque (PREVENT, NCT02316886) trial randomized 1,606 patients with functionally non-significant but vulnerable lesions—identified by intracoronary imaging—to PCI plus OMT or OMT alone. Preventive PCI significantly reduced 2-year MACE compared with OMT alone (0.4% vs. 3.4%; − 3.0% [95%CI, − 4.4 to − 1.8]; *p* = 0.0003), and the number-needed-to-treat was 45.4 to prevent one MACE over 2 years [52].

Several ongoing trials are further evaluating vulnerability-guided preventive PCI. The INTERCLIMA trial (NCT05027984) randomizes patients with intermediate non-culprit lesions to OCT-based or physiology-based strategies, with a 2-year primary endpoint of cardiac death or non-fatal spontaneous target vessel MI. The COMBINE-INTERVENE trial (NCT05333068) enrolls multivessel disease patients and compares an OCT plus fractional flow reserve (FFR) strategy with FFR-only guidance, with a 2-year composite endpoint of cardiac death, MI, or clinically driven revascularization. In STEMI patients with multivessel disease, the VULNERABLE trial (NCT05599061) tests whether PCI plus OMT improves outcomes compared with OMT alone for functionally non-significant lesions with OCT-defined TCFA. The primary endpoint is 4-year target vessel failure [53]. The DEBuT-LRP trial (NCT04765956) investigates drug-eluting balloon treatment of lipid-rich, non-culprit plaques (maxLCBI_4mm_ > 325) in ACS patients, with the primary endpoint being the change in maxLCBI_4mm_ at 9 months [54].

Importantly, not all high-risk plaque features predict long-term events, while stent-related complications can continue to accumulate over time. Long-term trials are needed to confirm whether preventive PCI offers greater protection than systemic therapy. Moreover, a vulnerable plaque reflects systemic atherosclerotic activity and increased risk of disease progression. Thus, even if local mechanical therapy is applied, systemic pharmacologic treatment remains essential to curb global atherosclerotic progression.

## Detection of Vulnerable Plaques by Non-Invasive Coronary Computed Tomography Angiography

While invasive intracoronary imaging offers detailed plaque characterization, its procedural risks, cost, and limited feasibility for whole-vessel or repeated assessments constrain its role in routine screening and follow-up—especially in primary prevention. To address these limitations, non-invasive cardiac imaging techniques for detecting high-risk plaques are gaining interest. Early identification may allow timely initiation of intensive pharmacotherapy and lifestyle interventions, improving preventive outcomes.

### Prognostic Value of high-risk Plaque Characteristics on CCTA

Coronary computed tomography angiography (CCTA) has emerged as a non-invasive modality for quantitative and qualitative plaque analysis [55, 56]. Compared with intracoronary imaging, CCTA allows whole-vessel visualisation and quantification of total plaque burden. Despite its spatial resolution being ~ 50 times lower than OCT and ~ 5 times lower than IVUS, precluding fibrous cap thickness or macrophage detection, CCTA-specific high-risk plaque (HRP) features have been defined—low-attenuation plaque (LAP), positive remodelling, napkin-ring sign, and spotty calcification—with strong prognostic validation [55–60] and correlation with OCT high-risk features [61]. In the PROspective Multicenter Imaging Study for Evaluation of Chest Pain (PROMISE, NCT01174550) trial [59], any HRP feature (LAP, positive remodelling, napkin-ring sign) was associated with a 2.7-fold higher MACE risk (HR 2.73, 95%CI 1.89–3.93) over 25 months. In (Scottish COmputed Tomography of the HEART Trial (SCOT-HEART, NCT01149590), LAP or positive remodelling was linked with a 3-fold higher risk of CHD death or non-fatal MI at 5 years (HR 3.01, 95%CI 1.61–5.63, *p* = 0.001) [58]. A meta-analysis of six studies with 2.3–8.2 years of follow-up confirmed prognostic significance of each HRP: LAP (HR 2.95, 95%CI 2.03–4.04), positive remodelling (HR 2.58, 95%CI 1.84–3.61), napkin-ring sign (HR 5.06, 95%CI 3.23–7.94), and spotty calcification (HR 2.25, 95%CI 1.26–4.04). Presence of ≥ 2 HRP features yielded the highest risk (HR 9.17, 95%CI 4.10–20.50) [57].

### Latest Advances in CCTA Technology

Recent technical advancements, including photon-counting CT, have improved CCTA resolution, potentially addressing previous limitations [62]. Artificial intelligence (AI) now enables rapid, semi-automated plaque quantification and characterization from CCTA [63–65], paving the way for its use in clinical practice. CCTA is also suitable for serial imaging to monitor disease progression and treatment response, though consistency in scanner and analysis settings is essential [55].

### Monitoring anti-atherosclerotic Treatment Effects by CCTA

Small RCTs have shown statins reduce non-calcified plaque volume on CCTA in patients with HIV [66, 67] and increase calcified plaque in post-MI patients [68] (Table 2). In a meta-analysis of 792 patients (2 RCTs, 10 observational studies, median follow-up 14.5 months), intensive statins reduced total plaque volume by 20.9 mm³ (95%CI − 31.2 to − 10.6, *p* < 0.001); moderate statins, by 1.7 mm³ (*p* = 0.69); and controls showed a 15.0 mm³ increase (*p* = 0.002). LAP volume significantly decreased (–5.8 mm³, 95%CI − 8.0 to − 3.7, *p* < 0.001), and calcified plaque increased (11.8 mm³, 95%CI 3.4 to 20.3, *p* = 0.006) with any statin [69] (Table 2). The objective of the Effect of Vascepa on Improving Coronary Atherosclerosis in People With High Triglycerides Taking Statin Therapy (EVAPORATE, NCT029226027) trial (*N* = 68) showed significant LAP volume reduction with icosapent ethyl vs. placebo in patients with elevated triglycerides despite statin therapy [70] (Table 2). Small observational studies reported increased plaque density and reduced remodeling index with PCSK9 inhibitors [71] and LAP volume reduction with low-dose colchicine [72] (Table 2), though these findings require validation in RCTs using conventional imaging endpoints. Ongoing studies—SCOT-HEART 2 (NCT03920176), DANE HEART 2.0 (NCT05677386), and TRANSFORM (NCT06112418)—are investigating whether CCTA-based care improves outcomes and alters plaque burden/composition compared with risk score–based care.

In summary, owing to its recent technical advances, CCTA may no longer remain the first-line anatomical imaging modality for ruling out obstructive CAD, for which it assumes a Class IA recommendation in current guidelines [46, 73], but may in the future also be acknowledged as an imaging modality for plaque burden quantification and characterization, tailored treatment decisions, and monitoring of anti-atherosclerotic treatment effects.

## Conclusions

The detection and treatment of vulnerable plaques play a pivotal role in improving the prognosis of patients with CAD. Intracoronary imaging modalities—including IVUS, NIRS, and OCT—remain the gold standard for identifying vulnerable plaques and evaluating the efficacy of therapeutic interventions. Lipid-lowering therapy is the first-line strategy for achieving plaque regression and stabilization. The degree of plaque volume reduction and fibrous cap thickening appears to be closely associated with on-treatment LDL-C levels. The potential of anti-inflammatory therapies to promote plaque stabilization, the clinical benefit of preventive PCI for non-obstructive vulnerable plaques, and the role of non-invasive imaging in allocating patients with high-risk lesions to advanced therapies remain areas of active investigation. Future research is expected to provide further insights into these strategies and to help refine a personalized, risk-guided approach to cardiovascular prevention.

ACS, acute coronary syndrome; AMI, acute myocardial infarction; aHR, adjusted hazard ratio; CCS, chronic coronary syndrome; CD, cardiac death; CHD = coronary heart disease, CI, confidence interval; HR hazard ratio; HRP = high-risk plaque, LAD, l eft anterior descending artery; LAP = low-attenuation plaque, maxLCBI_4mm_, maximum Lipid Core Burden Index in a 4 mm segment; MACE, major adverse cardiovascular event; MI, myocardial infarction; minFCT, minimum fibrous cap thickness; MLA, minimum lumen area; PB, plaque burden; SAP, stable angina pectoris; VH-TCFA, virtual histology intravascular ultrasound-derived thin-cap fibroatheroma.

ACS, acute coronary syndrome; AMI, acute myocardial infarction; ASCVD = atherosclerotic cardiovascular disease, CCS, chronic coronary syndrome; CCTA = coronary computed tomography angiography, LAP = low-attenuation plaque, maxLCBI_4mm_, maximum Lipid Core Burden Index in a 4 mm segment; LDL = low-density lipoprotein, LM = left main, FDG-PET = fluorodeoxyglucose positron emission tomography, minFCT, minimum fibrous cap thickness; HIV = human immunodeficiency virus, HU = houndsfield unit, IMT = intima-media thickness, MLA, minimum lumen area; NSTEMI, non-ST-elevation myocardial infarction; PAV, percent atheroma volume; SAP, stable angina pectoris; STEMI, ST-elevation myocardial infarction; TAV, total atheroma volume, VP = vulnerable plaque, *Log adjusted plaque volumes mm^3^.

## Key References

- Raber L, Ueki Y, Otsuka T, Losdat S, Haner JD, Lonborg J, et al. Effect of Alirocumab Added to High-Intensity Statin Therapy on Coronary Atherosclerosis in Patients With Acute Myocardial Infarction: The PACMAN-AMI Randomized Clinical Trial. JAMA. 2022;327(18):1771-81.
  - Findings from this study suggest the benefit of intensive lipid-lowering therapy with PCSK9inhibitor on plaque regression and stabilization.

- Park SJ, Ahn JM, Kang DY, Yun SC, Ahn YK, Kim WJ, et al. Preventive percutaneous coronary intervention versus optimal medical therapy alone for the treatment of vulnerable atherosclerotic coronary plaques (PREVENT): a multicentre, open-label, randomised controlled trial. Lancet. 2024;403(10438):1753-65.
  - Findings from this study suggest the benefit of preventive intervention for the vulnerable plaque.

## Data Availability

No datasets were generated or analysed during the current study.
